# The Impact of the COVID-19 Pandemic on Dentistry and Dental Education: A Narrative Review

**DOI:** 10.3390/ijerph19052537

**Published:** 2022-02-22

**Authors:** Ancuta Goriuc, Darius Sandu, Monica Tatarciuc, Ionut Luchian

**Affiliations:** 1 Department of Biochemistry, Faculty of Dental Medicine, “Grigore T. Popa” University of Medicine and Pharmacy, 700115 Iasi, Romania; ancuta.goriuc@umfiasi.ro; 2 Faculty of Dental Medicine, “Grigore T. Popa” University of Medicine and Pharmacy, 16 Universitatii St., 700115 Iasi, Romania; darius-valentin.lr.sandu@students.umfiasi.ro; 3 Department of Dental Technology, Faculty of Dental Medicine, “Grigore T. Popa” University of Medicine and Pharmacy, 700115 Iasi, Romania; 4 Department of Periodontology, Faculty of Dental Medicine, “Grigore T. Popa” University of Medicine and Pharmacy, 700115 Iasi, Romania; ionut.luchian@umfiasi.ro

**Keywords:** SARS-CoV-2 infection, dental healthcare, dental training, teledentistry, pandemic, dental aerosols

## Abstract

Dentists and dental staff have an increased risk of airborne infection with pathogens such as SARS-CoV-2 since they are exposed to high levels of droplets and aerosols produced during specific dental procedures. Hence, new guidelines such as patient screening and temperature control, air purification, space, surface and hand sanitizing and the use of protective equipment and physical barriers have been successfully implemented. In addition, the use of teledentistry has expanded considerably in pediatric dentistry, orthodontics, oral medicine and periodontics in order to address oral and dental health issues during the COVID-19 pandemic while minimizing virus transmission. Thus, teleconsultation, telediagnosis, teletriage, teletreatment and telemonitoring have emerged as valuable tools not only in the delivery of care, but also in the academic and research training of dental health professionals. This narrative review summarizes the current literature on the impact of the pandemic on dental care, dental staff and dental education, with an emphasis on how newly emerging protocols and technologies can be successfully utilized as integral parts of various branches of the dental practice and their future implications without compromising patient care.

## 1. Introduction

According to data provided by the World Health Organization (WHO), the new coronavirus, SARS-CoV-2, had caused 5,493,846 deaths globally by 10 January 2022, of which approximately 20% were recorded in the USA. Multiple factors are responsible for differences in contamination and mortality due to SARS-CoV-2 between countries. These include the implementation of domestic policies to control the spread of infection, vaccination, population density, comorbidities and the proportion of the ageing population, to name a few [1]. This has led to a significant variation in the degree of infectiveness and mortality (Table 1 and Table 2) [2].

Among medical practitioners, dentists and dental staff have an increased risk of being infected with airborne pathogens such as SARS-CoV-2 because they are always exposed to droplets and aerosols produced during specific treatment procedures. Transmission may occur due to the inhalation of droplets and aerosols from an infected individual or by direct contact with mucous membranes, oral fluids and contaminated instruments or surfaces. To evaluate the effects of intraoral and extraoral aspiration on the spread of infection during dental treatments, the bacterial colonization of droplets and aerosols was evaluated following simulations of scaling by the dentist and dental hygienist in three healthy volunteers. Extraoral aspiration has been shown to reduce the production of droplets and aerosols, and since it is restricted to the left and back of the dental chair, right-handed operators could perform treatment with relatively low contact with the pathogens. This study suggests that both aspiration methods were effective; however, extraoral aspiration was more effective in reducing the number of droplets and aerosols compared to intraoral aspiration or a lack of aspiration [3]. Furthermore, it has been shown that saliva represents a potential source of contamination for many patients. This aspect is of critical importance in public health management, not only for SARS-CoV-2, but also for other pathogens, considering the high rate of exposure to saliva by dental professionals [4]. 

**Figure 1:**
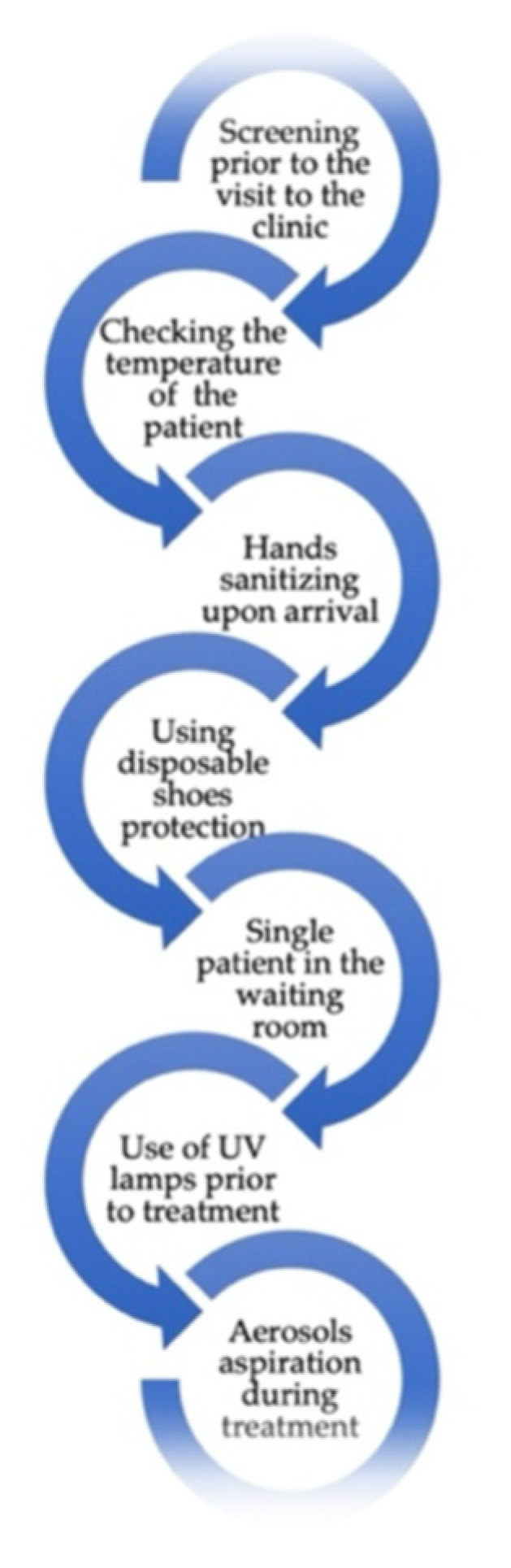
Safety protocol for dental patients during the COVID-19 pandemic.

Since COVID-19 is primarily spread through droplets and aerosols, it could reasonably be assumed that dentistry might be among the professions with the highest mortality rate [1]. However, when the number of deaths was examined in England and Wales between March and December 2020, there was no evidence of a higher mortality rate among dentists caused by COVID-19. This led to the conclusion that the low infection rate of dentists might be due to the rigorous safety protocols implemented. The American Dental Association (ADA), as well as most European dental organizations, recommends patient prescreening before visiting the clinic, allowing only one patient at a time in the waiting room, measuring staff and patients’ temperatures, hand washing and sanitizing, access to sanitizers for patients, disinfection of surfaces, personal protection equipment for the medical team, disposable shoe covers for patients, use of UV lamps and other air purifiers and high-efficiency aspiration during treatments (Figure 1) [1,5]. For example, Butera et al. suggested the use of the bio-inspired systems in nonsurgical periodontal treatment in order to reduce the risk of bacteremia and aerosol generation and improve clinical, microbiological and immunological parameters by decreasing bacterial load [6].

This qualitative, narrative review summarizes the most recent literature on the effects of the COVID-19 pandemic on dental practice and dental education, as well as the use of teledentistry in the delivery of oral and dental care to avoid virus contamination. Research and review papers were identified and selected using Scopus, PubMed and Web of Science scientific databases. Commentaries, letters and in vitro studies were excluded from the analysis. The paper describes in a comprehensive and critical manner, the effects of COVID-19 pandemic on the delivery of oral and dental care and dental education and its impact on current and future dental practice. 

## 2. COVID-19 and the New Approach to Dental Healthcare

Teledentistry has been defined as “the remote practice of dentistry by oral health professionals, within the limits of their practices, via the use of information and communication technology” [6]. Its objectives should not depart from those of in-person care, and may include diagnosis, prevention and post-treatment monitoring, specialist advice, treatment, prescription, referrals and other practices. Approximately 80% of dentists have adopted precautionary recommendations and modified them according to the type and particularities of each dental treatment [7]. For example, to increase the safety of the working team, a recent study showed that approximately 30% of dentists wore additional protective equipment, applied sanitation and ventilation procedures beyond those recommended by the guidelines and local health authorities, preferred to treat infected patients or those suspected of infection at the end of the working day and used an FFP3 mask during treatment. Approximately 78% of dentists replaced the FFP2 mask after eight hours of use, even when treating non-contaminated patients, and 62% covered the FFP2 mask with an FFP1 surgical mask [7]. 

Furthermore, 89% of dentists recommended oral rinses with solutions based on hydrogen peroxide and chlorhexidine before commencing therapeutic procedures. The combination of hydrogen peroxide with chlorhexidine solutions has been shown in vitro to be more effective than either solution alone in preventing transmission of SARS-CoV-2 [7]. Other studies have shown a decrease in salivary viral load after a 30 s mouth and oropharynx gargle with 15 mL of 1.5% or 3% hydrogen peroxide solution or 0.12% chlorhexidine solution [8]. Likewise, brief (30 s) rinses with 0.2%, 0.4% or 0.5% povidone–iodine (9 mL) or 0.05% cetylpyridinium chloride (15 mL) have been proven to be effective. Similar effects were obtained with cetrimide rinses in oncologic patients. However, the degree to which these solutions are effective in preventing or decreasing SARS-CoV-2 contamination risks, particularly in vulnerable populations, still need to be examined [9]. 

Although SARS-CoV-2 has a predominantly airborne transmission, salivary contamination can be controlled much easier in dental offices. For example, recent studies that examined increasing suction capacity by using a large volume of air (150 mm Hg or 325 L/min) suggest that this measure may be sufficient to eliminate viral contamination [7]. To further increase safety at work, Italian dentists have adopted, as a preventative measure, ventilation of dental treatment rooms after examination of each patient, regardless of the dental treatment performed. In rooms with poor mechanical ventilation, portable air filters with a high-efficiency particulate air filter (HEPA) have effectively reduced aerosol accumulation and accelerated aerosol removal [10]. Taken together, these studies show that new measures put in place in dental offices significantly mitigate the risks of SARS-CoV-2 contamination, and the risk of contracting COVID-19 in the dental office is relatively low [11].

## 3. Dental Public Health Issues during the COVID-19 Pandemic 

A recent study conducted in Italy showed a state of normalcy in dental practices after the initial wave of COVID-19 pandemic. Since its onset, Italian dentists have experienced high levels of anxiety and stress, mainly due to the rapid spread of SARS-CoV-2 at the national level and the need for rapid adaptation to the new health standards in dental offices [7]. Approximately 80% of Italian dentists resumed their regular activity after the first quarantine. However, there were some geographical differences due to the evolution of the virus over time. For example, the reopening rate of dental offices ranged from 36% in the United Kingdom to 47% in Palestine, while in Italy and the USA this figure reached 99%. Approximately 80% of dentists have adopted preventative measures and adapted them to specific dental treatments [7]. Notwithstanding these changes, dental offices incurred significant financial losses of over 70% due to the COVID-19 pandemic. In a survey conducted by the British Dental Association, 70% of dental clinics reported that they could only remain viable and maintain their usual number of employees for up to three months [7].

## 4. Pre-, during and Post-Pandemic Particular Aspects of Dental Treatments

A recent study highlighted the changes in the spectrum of procedures performed before and during the pandemic. For example, during the COVID-19 pandemic, the number of conservative procedures, such as coronal restorations or root canal fillings, decreased significantly, while the percentage of surgical procedures increased significantly. In the following months, the decrease in the number of patients was offset by an increased number of procedures per visit [12,13]. Likewise, several changes were recommended when performing various treatment procedures. For example, the mechanochemical treatment of carious lesions was carried out using hand tools instead of rotary ones. Similarly, for periodontal treatments, manual scaling was chosen over ultrasonic scaling. In cases of symptomatic irreversible pulpitis, biological methods, such as pulpotomy or pulpectomy, were recommended as much as possible [14]. On the other hand, in patients with extensive destruction of the hard dental tissue accompanied by severe pain, it was necessary to opt for the extraction of the affected tooth. Thus, it was possible to reduce the risk of infection, shorten treatment time and minimize repeated visits. In the case of excessive bleeding, multiple extractions or other oral surgeries, resorbable sutures were preferred. Specific interventions have been performed in pediatric patients to reduce aerosol-generating procedures and use non-invasive or minimally invasive methods. For example, fissure sealing, the topical application of varnishes and resin infiltration using the ICON method, were chosen in order to stop the evolution of non-cavitary carious lesions. At the same time, to minimize virus transmission and contamination, there was an increase in the number of certain procedures, such as indirect capping, atraumatic restorative treatment, provisional therapeutic restorations, the Hall technique and the application of diamine silver fluoride [13].

## 5. Teledentistry and COVID-19

Telemedicine has proven to be an effective tool in mitigating some of the effects caused by the imposition of restrictive measures during the COVID-19 pandemic. Several authors have suggested that the lack of coherence in the implementation of telemedicine as a solution for continuous medical education is one reason for the absence of uniform protocols for aerosol-generating procedures [14]. For example, dentists in the UK changed the way they approached clinical cases and developed a triage system using remote consultations. The treatment was limited to advice, analgesia and first-line antimicrobial therapy, with the goal of reducing the risk of transmission. COVID-19-positive patients, confirmed by RT-PCR, were directed to in-person treatment only in emergency dental centers, which have been previously authorized for this purpose [1].

**Figure 2:**
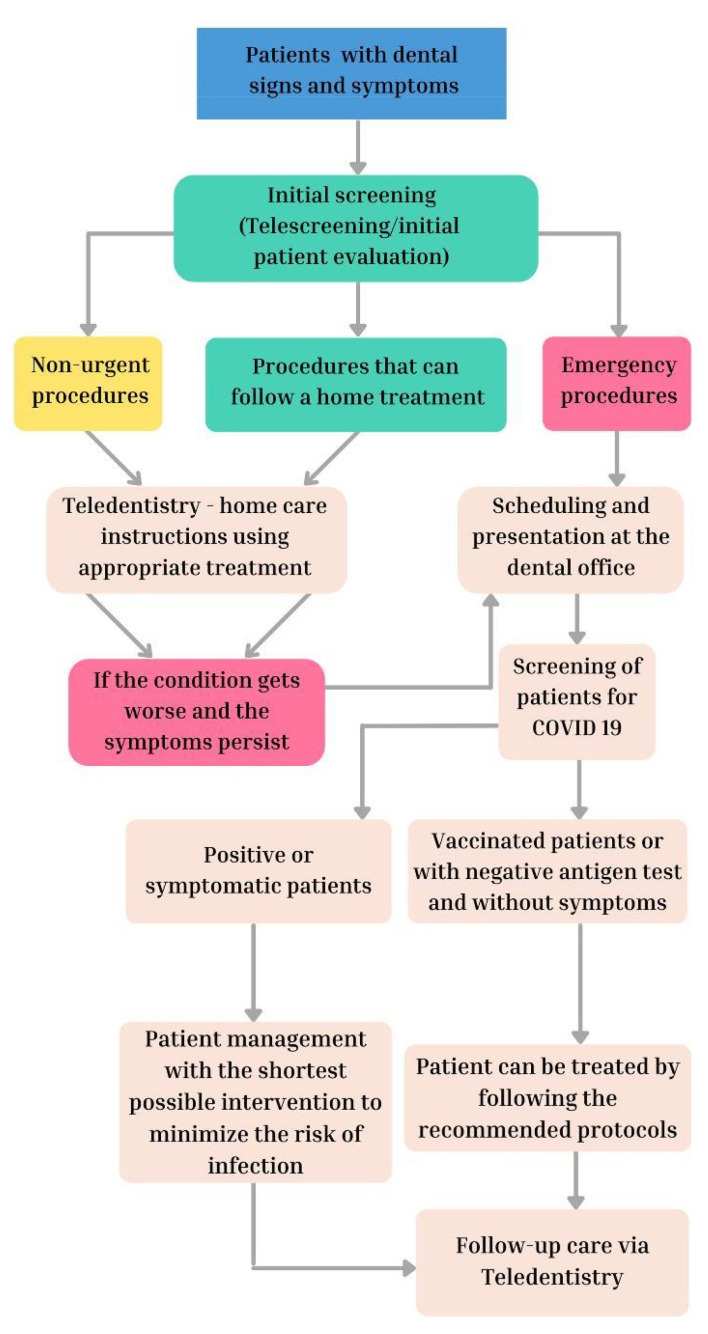
Management of dental protocols during COVID-19 pandemic.

Teledentistry has been increasingly used by dental schools during the pandemic. Although there are regional differences in isolation policies, the severity of the outbreak and the availability of resources have greatly impacted the functioning of dental schools during the pandemic, although the responses of dental institutions to the COVID-19 pandemic show some similarities. For example, distance learning was the only alternative to theoretical dental education in many institutions. While e-learning already existed, it evolved and expanded as a result of the COVID-19 pandemic, with the use of synchronous online teaching methods when interacting with participants [15]. In Italy, for example, telemedicine played a key role in reducing the spread of COVID-19 from the start of the pandemic. A number of teledentistry platforms such as OloHealth^®^ have emerged since 2019 and are dedicated to the prevention and management of oral malignant disorders in addition to improvement of oral health, in order to reduce unnecessary travel and loss of productivity [16]. Likewise, teledentistry has been used successfully when treating patients with more complex oral pathologies by carrying out photographic teleconsultations for the first visits and for subsequent evaluations, thus ensuring a good remote patient management (Figure 2). After an adequate anamnesis by videocall and photographic evaluations during the first visit, patients were followed up with remote evaluations of their pathologies, such as fungal infections, dry mouth syndrome, sialolithiasis, traumatic ulcers, third molar pericoronitis and others [17].

Telemedicine was further used in cases that would normally require clinical examination in order to distinguish between potentially malignant lesions from those that were truly malignant and necessitated immediate attention. This allowed dentists to keep patients with precancerous lesions, osteonecrosis of the jaw associated with medication and autoimmune diseases under control by comparing recently received photos with the last photos taken at the dental clinic. For these pathologies, clinical changes were evaluated to determine the risk of malignant processes and manage possible recurrences, infections, pain and stability of lesions [16]. For example, Machado et al. emphasized the importance of oral telediagnosis when examining a 49-year-old female patient with controlled diabetes and symptomatic pinkish nodular lesions affecting the buccal mucosa, associated with purple spots on the skin. The dentist took photos with a short description using the WhatsApp platform and recommended a hematological examination based on idiopathic purpura. Severe thrombocytopenia was confirmed, and the patient was referred to the hospital for specialized systemic treatment with steroid medication [18]. When teledentistry is not available or cannot be used, saliva tests can be employed as a solution for screening patients with minimal physical contact given the strong link between salivary diagnosis and oncologic pathology [19]. 

Notwithstanding, teledentistry appears to be a promising tool in the remote management of patients requiring non-surgical or surgical treatment, especially by reducing costs and waiting times [15]. To this end, patients that need regular treatment for chronic conditions, geriatric patients or those with special needs, and patients living in remote, less-accessible areas can benefit the most from teledentistry. This can lead to reduction in the number of visits to the dental office, shorter waiting times, decreased no-show appointments, and reduced unnecessary exposure of healthy patients. The efficacy of teledentistry has mostly been studied in pediatric dentistry, oral medicine, orthodontics and periodontics for several procedures and with various degrees of success. For teledentistry to be effective, it requires an educated patient not only familiar with the new digital technologies (i.e., taking high-quality intraoral digital images, data storage, virtual communications using various apps, etc.) but also with basic dental knowledge [20]. For example, knowledge of the management of orthodontic appliances or teeth eruption, which represent a significant portion of emergency visits, can reduce office visits by approximately 20 percent. Fixing a loose or poking orthodontic wire or appliance, smaller fractures or tissue trauma can all be handled by teledentistry and avoid in-person visits. Indeed, some studies have shown that nearly half of all dental emergencies can be managed utilizing teledentistry. However, some of these procedures such as chronic pain, dental caries, severe trauma, fractures and orthodontic treatments would need to be evaluated frequently to avoid unnecessary delays that may lead to later complications such as infections. Not all dental practices and specialties are prepared or have the expertise for successful use of teledentistry, and it requires significant resources and infrastructure. For this reason, there is a wide range of teledentistry applications and its use varies across countries, regions, dental specialties and offices. Irrespective of these differences, the most important advantage lies in triaging patients, reducing the number of site visits and, in doing so, reducing exposure to pathogens such as SARS-CoV-2 [21].

In addition to the various devices that can be used in telemedicine, instant messaging applications have become increasingly popular for better communication between doctor and the patient. For example, WhatsApp-based teleradiology consultation proved to be an effective tool for interpreting X-rays with different dental pathologies [22]. Furthermore, dental telemedicine can be successfully applied during the follow-up of patients who have undergone oral and maxillofacial surgery, although more work is required to determine patients’ compliance and doctors’ attitudes towards integrating remote dentistry in the standard protocols of telemedicine [23].

## 6. The Impact of COVID-19 on Dental Staff

It is well established that COVID-19 had a significant impact on nurses working in the field of dentistry, which affected the quality of medical services. Chronic diseases, immunodeficiencies, the risk of SARS-CoV-2 infection, working in a private environment and family responsibilities associated with financial risks caused by the pandemic have all contributed to significant increases in anxiety, burn-outs and other mental health disorders [24]. This necessitates collective actions at the government level and a set of measures that can contribute both to the prevention and treatment of these manifestations [24,25]. Among dental staff, dental hygienists have been greatly impacted by the pandemic given the high risk of occupational contamination via aerosol loading due to their work in the maintenance of periodontal health and prevention of dental diseases. Although there are differences in the application of working protocols of prophylaxis, for assistants working in the private sector compared to those working in the public health system [24], the emphasis has been on minimizing the use of aerosol-generating procedures [25]. The existence of protective equipment for dental teams, an adequate infrastructure as well as the correct management of patients, all contributed to an increase in trust and safety within medical teams [24]. For example, a recent study conducted in the Czech Republic showed that well-chosen anti-epidemic measures adopted by dental professionals can reduce occupational infection risks associated with SARS-CoV-2 [26]. On the other hand, other studies examining the knowledge, attitudes and practices of the Turkish pediatric dentists, showed a satisfactory level of knowledge regarding COVID-19 prevention, although infection control measures could have been better implemented [27].

## 7. The Impact of COVID-19 on Dental Academic Environments

### 7.1. Emotional and Psychological Effects

Academics working in the field of dentistry have been subjected to a high level of stress during the COVID-19 pandemic, not only related to teaching and research activities but also to concerns related to the possibility of contaminating their family members [28]. This triggered an immediate and acute need for developing and implementing psychological support measures to reduce the level of mental stress among members of the academic staff. For example, Balkaran et al. recommended the use of meditation, specialized counselling and holding seminars for health promotion as therapeutic measures [28]. Similarly, a plethora of measures have been proposed for dental students, given the critical role of mental health in the educational evolution and behavioral development of dental students preparing for medical careers [29,30]. Chronic cardiovascular diseases, smoking and being female, as well as the economic impact of SARS-CoV-2 on the dental profession, have been shown to negatively affect the psychological status of dental students [24]. 

The COVID-19 pandemic has led to an increase in negative emotional states among students. Students were the population group most affected by the pandemic, showing an increased prevalence of stress, anxiety and insecurity [31]. Female students were found to be affected more than males, with a high risk for developing depression and negative emotional states, which was associated with an increase in leisure time and decreased physical activity [30]. Therefore, examining these factors can play a key role in developing public health policies to minimize the psychological impact among future dentists.

### 7.2. Quality of Dental Education

The COVID-19 pandemic has considerably affected the quality of dental students’ training. Dentistry is a scientific–educational field which combines theoretical concepts and principles with the acquisition of practical skills. In the absence of a mandatory residency program, basic dental education requires sufficient preclinical and clinical training to ensure an adequate level of competence for future professionals in the field [32]. While distance learning could be a commonly adopted strategy for higher education in various fields, a unique challenge for dental education is the dependence on the requirements of clinical experience to achieve minimal competency in performing dental treatments [33]. Since many dental procedures produce considerable amounts of aerosols and droplets, many routine and elective dental treatments were suspended during the pandemic, thus affecting the training of dental students [1].

### 7.3. Dental Research

The COVID-19 pandemic has greatly impacted research in the dental field. In some cases, having limited access to patients delayed and even compromised the results of clinical trials and restricted the implementation of new ones. For example, saliva and crevicular fluids are valuable diagnostic tools in dental medicine [34,35]. Diagnostics based on salivary matrix metalloproteinases (MMPs) that can successfully quantify periodontal inflammation should be cautiously used because of potential contamination risks [35,36,37]. To overcome these hazards, other methods have been employed, such as the finite element method (FEM), which uses mathematical models to simulate clinical reality and does not involve patient contact [38,39]. FEM proved to be an extremely useful and reliable alternative option during the COVID-19 pandemic by providing optimal prognoses and validating different protocols of treatment in various dental specialties, such as periodontics, orthodontics or prosthodontics [39,40,41,42,43].

## 8. Perspectives and Limitations

The COVID-19 pandemic has affected and still continues to significantly impact the delivery of dental healthcare due to changing clinical protocols and adapting them in order to minimize contamination risks. Teledentistry has expanded its initial scope and, when correctly implemented, could be an effective tool, but should be considered as a complementary means rather than an alternative to on-site, conventional treatments that are based on the principle of personalized medicine. Teledentistry can offer tremendous benefits in the delivery of some applications, while it can be limited in others. For example, pre- and post-operative counselling, education and care, nutrition advice and quick access to images of oral cavities through user-friendly imaging devices accessible to patients can all be performed via teledentistry. On the other hand, there are many challenges associated with teledentistry. These are primarily related to the lack of guidelines, standardization and scientific validation of teledentistry procedures and tools used in addition to issues related to data security and privacy. Other constraints are related to the inability to perform a clinical tactile exam, lack of direct contact with the patients, risk of misdiagnosis, lack of technological infrastructure, poor access to the Internet, lack of hardware, low information technology literacy, resistance to new technologies, and a lack of training and consumer awareness. There is no “one-fit-all” solution to overcome these challenges, and as the field evolves, new creative models will be developed in order to fit particular scenarios. Irrespective of the model used, the patient should be provided with the same quality of dental care as performed in the clinic. As a minimum, it should meet the following criteria: provide easy access to all populations, including the underserved, provide oral health delivery, including specialty care in a timely manner, and be sustainable, affordable and time effective. There are several limitations of this narrative review that include the inherent lack of quantitative analyses of published studies and being prone to biases. Notwithstanding, teledentistry proved to be useful in enhancing communication with, and treatment of, various categories of patients, such as those from nursing homes and prisons, and it is cost and time efficient. It is readily accessible, user friendly, and will no doubt continue to be expanded to new areas of dentistry and remote dental services, even after the COVID-19 pandemic. Moving forward, teledentistry will play a significant role in dental education and curriculum delivery by using technological advances in offering the skills required to maintain the quality of care while minimizing disease transmission. Finally, there is a need for more long-term comprehensive studies evaluating the impact of teledentistry in prevention, clinical outcomes and delivery of treatment across all branches of dentistry, as well as in developing distant training protocols in order to provide dental education in a safe environment.

## 9. Conclusions

Dental staff, academic personnel, dental students and dental researchers were severely affected by the COVID-19 pandemic, which led to a decrease in the quality of care, clinical work and practical training. Despite its limitations, teledentistry has become a critically important tool during the COVID-19 pandemic in mitigating the risks of virus contamination and transmission. Overcoming challenges in adopting teledentistry by improving patient and management tools via new technologies coupled with innovations in dental engineering and equipment to minimize aerosol-transmitted pathogens will, no doubt, find the dentistry world better prepared to withstand the negative impact of a potential future pandemic. Thus, future research should focus on improving the quality and reliability of teledentistry in order to eliminate current technological errors and further integrating it as a complementary option in dental healthcare systems worldwide.

## Figures and Tables

**Table 1:** SARS-CoV-2 infection rate by August 2021 [2].

SARS-CoV-2	Percentage
United States	18.19%
India	15.22%
Brazil	9.65%
Russia	3.17%
France	3.11%
United Kingdom	3.06%
Spain	2.25%
Romania	6.87%
Average	7.69%

**Table 2:** SARS-CoV-2 cases and mortality rate by 10 January 2022 (https://coronavirus.jhu.edu/data/mortality (accessed on 26 August 2021)).

Country	Cases Confirmed	Deaths	Case Fatality (%)
Peru	2,358,685	203,019	8.6%
Brazil	22,529,183	620,251	2.8%
Belgium	2,231,686	28,459	1.3%
Italy	7,436,939	139,038	1.9%
Mexico	4,125,388	300,334	7.3%
United States	60,074,429	837,594	1.4%
United Kingdom	14,563,769	150,634	1.0%
Ecuador	559,950	33,699	6.0%
Romania	1,844,537	59,011	3.2%
Spain	7,164,906	89,934	1.3%
Portugal	1,499,976	19,029	1.3%
France	12,218,022	126,427	1.0%
South Africa	3,526,054	92,453	2.6%
Iran	6,206,405	131,878	2.1%
Russia	10,470,006	309,787	3.0%
Greece	1,507,616	21,394	1,4%
Austria	1,339,421	13,848	1.0%
Germany	7,553,743	114,033	1.5%
Average	970,464,876	163,519,323	2.70%
